# Which Concept of Concept for Conceptual Engineering?

**DOI:** 10.1007/s10670-021-00447-0

**Published:** 2021-09-17

**Authors:** Manuel Gustavo Isaac

**Affiliations:** Arché Philosophical Research Centre, University of St Andrews, 17–19 College Street, St Andrews, Fife, KY16 9AL Scotland

## Abstract

Conceptual engineering is the method for assessing and improving our concepts. However, little has been written about how best to conceive of concepts for the purposes of conceptual engineering. In this paper, I aim to fill this foundational gap, proceeding in three main steps: First, I propose a methodological framework for evaluating the conduciveness of a given concept of concept for conceptual engineering. Then, I develop a typology that contrasts two competing concepts of concept that can be used in conceptual engineering—namely, the philosophical and psychological ones. Finally, I evaluate these two concepts of concept using the proposed methodological framework and I show that, when it comes to making conceptual engineering an actionable method, the psychological concept of concept outclasses its philosophical counterpart on all counts. This provides a baseline from which the concept of concept can be further improved for the purposes of conceptual engineering.

## Introduction

Conceptual engineering is the method for assessing and improving our concepts. However, little has been so far written on how best to conceive of concepts for the purposes of conceptual engineering.^1^ So little, in fact, that the current situation is still aptly characterized as follows:

There’s of course already a smorgasbord of options for how to think about concepts […]. However, and this is the strange part, those who talk of conceptual engineering as operating on concepts don’t start by making choices on this smorgasbord. They often just talk about ‘concepts’, their engineering, and then leave it at that. (Cappelen 2018: 141)

Or, more recently, by hinting at a possible explanation:

Proponents of th[e] standard account of conceptual engineering tend to say very little about *what concepts are*. I suspect this is because they don’t think much *needs* to be said about this. Philosophers speak of concepts all the time […]. Proponents of the standard account of conceptual engineering appear to think that there is no problem simply co-opting this ‘concept talk’, trusting that philosophers, at least, will know how to interpret it […]. (Deutsch 2020a: 4)

The aim of this paper is to fill this foundational gap, which concerns the very subject matter of conceptual engineering. I will proceed in three main steps. After briefly presenting my understanding of conceptual engineering (Sect. 2), I will propose a methodological framework for evaluating the conduciveness of a concept of concept to the purposes of conceptual engineering (Sect. 3). Then, I will develop a typology that contrasts two competing concepts of concept that can be used in conceptual engineering: The philosophical and the psychological concepts of concept (Sect. 4). Finally, I will compare these two types of concepts of concept, evaluating them with the proposed methodological framework (Sect. 5). After weighing some of the possible objections to my proposal (Sect. 6), I will conclude that the psychological concept of concept, in comparison to the philosophical one, has better prospects for making conceptual engineering actionable—that is, capable of being effectively applied to specific case studies (Sect. 7).

## The Basics

At first glance, conceptual engineering offers an updated take on philosophical methodology as consisting of the study of our concepts.^2^ A key feature of this renewed approach is its normative agenda: Conceptual engineers aim to prescribe what concepts we *should* have and use, rather than simply describe the concepts we *do* have and use. Although there is no unifying framework for conceptual engineering research, current work in the field shares several core principles, albeit tacitly.^3^

Firstly, concepts are usually treated as representational devices that serve cognitive functions for the execution of some cognitive tasks—that is, tasks whose execution requires the exercise of some cognitive competences. For instance, how the concept of sexual harassment enables its victims to make sense of the offensives they have experienced in a hostile work environment, or how the concepts of the GDP metrics help to measure a country’s economic progress. Secondly, it is typically assumed that better concepts lead to better ways of thinking, speaking, and behaving. For instance, one may consider that the concept of sex has been aptly replaced, in the social context, by that of gender in order to make sense of and fight against the many persistent inequalities of our social reality. Thirdly, it is generally believed that our conceptual apparatus can be (re-)engineered, and for the better.

In addition to these shared theoretical principles, the way in which conceptual engineering is applied as an ameliorative method commonly follows a two-step process. First, there is an assessment phase during which the quality of a conceptual device is typically measured in terms of its functional efficacy—that is, its fitness for its actual or intended function.^4^ Here the goal is to identify improvable features (such as deficiencies to fix) of a given conceptual device in terms of how well it fulfills its actual or intended function. Importantly, during this phase, the function of a concept itself may be found to be detrimental in some respect and thus in need of being revised or replaced by some new function (Burgess and Plunkett 2013b; Nado 2020a; Plunkett and Sundell 2013; Thomasson 2017, 2020); for instance, Haslanger (2000) has advocated for repurposing the extant concept of woman so that it challenges the many persistent gender-based inequities of our social world, instead of tracking females according to their biological sex.^5^ Then comes an improvement phase in which strategies for improving the conceptual device under consideration (such as for fixing its deficiencies) are prescribed, with a view to determining whether and how a given concept should be used. Importantly, both of these phases pertain to the normative dimension of conceptual engineering and depend on some preliminary descriptive work that generates the data to process. And, because conceptual engineering never takes place in a void, treating the descriptive phase with importance is critical for the whole enterprise, while ignoring it might lead to ‘disimproving’ things (cf. footnote 5).^6^

In light of this, conceptual engineering can be understood as a metaphilosophical movement that aims to normatively ameliorate the cognitive quality of our conceptual devices in terms of their functional efficacy.^7^ It is worth adding to that characterization that conceptual engineers never see conceptual amelioration as an end in itself, but rather, in terms of cognitive efficacy, as a means of changing how we reason, communicate, and behave toward each other, with a wide scope and a strong impact on the whole of our cognitive life. This was eloquently articulated in a seminal article by Burgess and Plunkett (2013a: 1096–1097):

Arguably, our conceptual repertoire determines not only what beliefs we can have but also what hypotheses we can entertain, what desires we can form, what plans we can make on the basis of such mental states, and accordingly constrains what we can hope to accomplish in the world. Representation enables action, from the most sophisticated scientific research, to the most mundane household task. It influences our options within social/political institutions and even helps determine which institutions are so much as thinkable. Our social roles, in turn, help determine what kinds of people we can be, what sorts of lives we can lead. Conceptual choices and changes may be intrinsically interesting, but the clearest reason to care about them is just that their non-conceptual consequences are pervasive and profound.

Such broad-spectrum ambitions, in terms of scope and impact on the whole of our cognitive life, ought to be seen as another distinctive feature of the conceptual engineering movement.

## The Bootstrapping Strategy

With the basics now in place, we can turn to the main problem that this paper addresses; namely, which framework is best suited to conceiving of concepts for conceptual engineering. To tackle this problem, the first step is to establish a provisional method for evaluating the conduciveness of a given concept of concept to the purposes of conceptual engineering. What we are searching for is some kind of adaptable set of step-by-step instructions for the amelioration of our conceptual apparatuses. Despite its notorious limitations, Carnap’s (1950) method for explicating concepts provides us with just such a framework.^8^ Carnapian explication squares well with both the evaluative and normative dimensions of the two phases of conceptual engineering (Sect. 2). Furthermore, it administers a set of weighted constraints that plausibly govern, in a context-sensitive way, any conceptual amelioration—though possibly with no “unique optimal trade-off” (Machery 2017: 217). On these grounds, we will draw on Brun’s (2016: 1226 ff.) reconstruction of it in the form of a ‘Recipe for explicating [concepts]’; and that reconstruction will then serve as a procedural template for further elaborating our provisional method for conceptual engineering, by comparison with the original Carnapian framework.^9^ This move can be referred to as the ‘bootstrapping strategy.’^10^

Carnapian explication, as rebooted for the purposes of conceptual engineering, can be presented as a three-step process, measured by three parameters, and regulated by two main requirements. The first step is conceptual regimentation, in which the target concept (a.k.a. the ‘*explicandum*’ in Carnap’s parlance) is clarified along two complementary dimensions. On the one hand, there is a relational dimension that specifies the system of concepts to which the target concept belongs, along with its status and function in relation to the other elements of that system. In the present case, the target concept is that of concept, while the target conceptual system is composed of all the different principles and dimensions that together make up the theoretical and methodological framework of conceptual engineering—for our purposes, such a relational dimension has thus already been clarified (Sect. 2). On the other hand, there is an elementary dimension (as opposed to the relational one), which focuses on the target concept of a given engineering project, irrespective of its relationships to other elements of its conceptual system, with the aim of determining its structural properties (e.g., its logical form, lexicological type, etc.) and the range of its relevant uses (e.g., via a taxonomy of its meaningful applications). In the case at hand, such elementary clarification of the concept of concept will allow us to distinguish between two competing concepts of concept that are suitable for the purposes of conceptual engineering (Sect. 4). Together, these two dimensions of the first step of our rebooted Carnapian explication correspond to the descriptive aspect of conceptual engineering as a method (Sect. 2), for they roughly consist of mapping “what possible and actual concepts there are” (Eklund 2021).

The second step of our rebooted Carnapian explication corresponds to the assessment phase of conceptual engineering. Thus, it consists of assessing the previously regimented target concept, including the identification of its improvable features, in terms of functional efficacy—in other words, for how well it fulfills its function as a cognitive device by being conducive to the execution of the cognitive task(s) that it is designed to enable. Three parameters, which are presented below in order of decreasing importance, are assessed. In Carnapian parlance, the first and most important of these parameters is *fruitfulness*, which means the suitability of the concept under consideration for formulating generalizations. In theoretical cases, fruitfulness can be characterized by predictive power and testability in the context of justification, and by the power to produce new knowledge in the context of discovery (Dutilh Novaes and Reck 2017). It therefore remains a rather malleable parameter, plausibly made up of a ‘cluster of criteria’ that may vary according to context and depending on the aim(s) of the explicator (Pinder 2017: 453 ff.). For our purposes, in a more applied fashion, it will be measured in terms of the scope and impact on our cognitive life that the available concepts of concept would ensure to conceptual engineering. Scope and impact, in turn, will be considered in light of the broad-spectrum ambitions of conceptual engineering; and, more specifically, in light of the pervasiveness and profoundness of the “non-conceptual consequences” that conceptual engineering is expected to have on the whole of our cognitive life (Burgess and Plunkett 2013a: 1097). Essentially, scope will concern the range of concepts that can be processed by the method of conceptual engineering being considered—from “the most sophisticated […] to the most mundane” (ibid.)—while impact will concern the way conceptual engineering may affect people’s minds and cognitive lives (more on this in Sect. 5).

*Exactness*, which may be glossed in terms of unambiguity, precision, and consistency, is the second of the three parameters for assessing a regimented concept in our rebooted version of Carnapian explication. In this case, consistency will mostly be contingent on the absence of constitutive principles that either contradict one another or conflict with otherwise uncontroversial facts (cf. Scharp and Shapiro 2017) and be detected by the presence of paradoxical issues. As for precision and unambiguity, they will be determined, respectively, by descriptive accuracy and descriptive adequacy. Both will depend on the quantity and quality of data available in the given framework, along with the framework’s theoretical purposes, but each will have different possible markers; namely, theoretical convergence in the case of precision and positive results in that of unambiguity. Finally, *simplicity*, which may be conceived in terms of both the definition and rules of use of the target concept, is the third and last parameter for assessing the regimented concept under consideration. In the framework of conceptual engineering, it will depend on obtaining an operationalizable concept of concept. And this, in turn, will bear crucially on, first, the possibility of detecting the need for conceptual amelioration, and second, on the possibility of the prescribed change being implemented.^11^ These three parameters (viz., fruitfulness, exactness, and simplicity) will serve in assessing two competing regimented concepts of concept, then in choosing the one most conducive to the purposes of conceptual engineering (Sect. 5).

The third and last of the three steps of our rebooted Carnapian explication coincides with the improvement phase of conceptual engineering (Sect. 2), and thus, this step consists of improving the target concept on the basis of its previous assessment. This improvement must meet two main requirements. First, it must preserve a certain degree of *similarity* with the original target concept. In line with the core theoretical principles of conceptual engineering (ibid.), similarity means here the continuity of the target concept’s function(s) with the conceptual system to which it belongs. In the present case, what will need to be preserved, then, is the explanatory function of the concept of concept for the cognitive functionalism that characterizes the conceptual system of conceptual engineering. Second, any improvement of the target concept must score better on the three parameters that make up the assessment phase of our methodological framework—namely, fruitfulness, exactness, and simplicity. These two requirements provide guidelines for how to improve the concept of concept that will be selected as the most conducive to the purposes of conceptual engineering (Sect. 7).

## The Mapping Project

By employing the rebooted Carnapian explication, the challenge can now be taken up of engineering the concept of concept at work in ‘conceptual engineering.’ The next step is to clarify the range of relevant uses of the concept of concept, for the purposes of conceptual engineering, via a typology of its meaningful applications (cf. Sect. 3). With this in mind, I shall now draw on a widely acknowledged, albeit not uncontroversial, dichotomy between two frameworks for theorizing concepts—namely, the philosophical and psychological—which further correlate with different approaches to and models of concepts (Johnston and Leslie 2012; Löhr 2018; Machery 2009; Peacocke 1992; Rey 1983, 1985). The controversial aspect of this dichotomy mostly bears on two things: First, on whether the distinction really exists; and second, on whether the two frameworks are compatible, and maybe even complementary to one another, or rather are mutually exclusive of one another.^12^ Since the purpose here is only to clarify the different options available when it comes to choosing the best framework for theorizing concepts as the subject matter of conceptual engineering (as opposed to, e.g., developing a putatively all-encompassing theory of concepts *per se*), none of these controversies affects the usefulness of the distinction as a heuristic. Be that as it may, both are described below in order to subsequently assess them with regard to their respective conduciveness for conceptual engineering (cf. Sect. 2). This step can be referred to, in accordance with Eklund (2021), as ‘the mapping project.’

Mainstream analytic philosophy of mind and language (e.g., Burge 1993; Fodor 1975; Peacocke 1992) forms the philosophical framework for theorizing concepts. Unsurprisingly, those who perceive conceptual engineering to be about concepts commonly adopt it.^13^ In the standard view, concepts are construed as semantic entities (roughly, the components of our thought contents) endowed with a ‘semantic structure’ (Margolis and Laurence 2010, 219) that serves a referential function. Construed thus, the semantic constituency of concepts becomes the focus. Typically, according to the so-called ‘classical model’ that still undergirds the philosophical approach, concepts consist of sets of separately necessary and jointly sufficient features, which are further characterized as semantically analytical and epistemically a priori. These constitutive features, in turn, are usually understood as being both independent from one another and of equal weight or importance within the conceptual structure they compose. In addition, they are usually meant to spell out the identity condition of that conceptual structure, along with its application criteria to the objects that fall within its extension. In light of this, the gist of philosophical theories of concepts can be captured in their attempt “to determine the condition under which people can have propositional attitudes about the object of their attitudes” (Machery 2010: 199), in order to ultimately deliver “a priori, analytic truths about the world” (Machery 2017: 209).^14^

The psychology of concepts (e.g., Murphy 2004) is the alternative framework available to conceptual engineering for theorizing about its subject matter. In contrast to its philosophical counterpart, little attempt has been made so far, with the exception of Isaac (2020), Koch (2020a), Machery (2017), to capitalize on this research field for the purposes of conceptual engineering. According to the psychological framework, concepts are viewed as cognitive entities endowed with a ‘processing structure,’ which “explains how concepts figure in various mental processes” (Margolis and Laurence 2010: 219). Thus, from this perspective, concepts are approached with a focus on their cognitive efficacy. Typically, according to the cognitive model that nowadays guides the psychological approach, concepts consist of structured bodies of information about some category of referents (viz., individuals, classes, substances, or event-types), which are retrieved and activated, or constructed, to play a causal/explanatory role in the cognitive processes that underlie most of our higher cognitive competences in relation to these referents (e.g., categorization, deduction, induction, analogy-making, action-planning, linguistic understanding, etc.) (cf. Machery 2009:12). These bodies of information are themselves now consensually taken to be realized by different, compatible, and/or complementary cognitive structures (e.g., exemplars, prototypes, and theories), and made up of different types of informational components (viz., information about single instances of category members in the case of exemplars, statistical information in the case of prototypes, and causal, functional, generic, and nomological information in the case of theories).^15^ In turn, these constitutive informational components are usually understood as being both interdependently influential on one another and of unequal weight or importance with respect to one another within the structured body of information they compose. With this in mind, we get to the gist of psychological theories of concepts in their attempt “to explain the properties of our higher cognitive competences” (Machery 2010: 199) through the formulation of “empirical propositions about the mind” (Machery 2017: 209).

Hopefully, this typology of the concepts of concept that are available to conceptual engineering yields a clear alternative between these two neatly regimented options—namely, the philosophical and psychological. These two options will serve as ideal types for choosing the best concept of concept for construing the subject matter of conceptual engineering.

## The Evaluative-Prescriptive Project

The next step to fill the foundational gap in conceptual engineering is to assess the regimented philosophical and psychological concepts of concept and then choose the one most conducive to the purposes of conceptual engineering. This step corresponds to the second of the three steps of the rebooted Carnapian explication (Sect. 3) and can be referred to, again in accordance with Eklund (2021), as ‘the evaluative-prescriptive project.’ Below, it will be carried out in three rounds, following our three recast Carnapian parameters of fruitfulness, exactness, and simplicity. The selected option will eventually provide us with a baseline from which to further improve the chosen concept of concept for the purposes of conceptual engineering (Sect. 7).

### Round One: Scope and Impact

In the Carnapian explication reboot, fruitfulness, exactness, and simplicity serve to assess the efficacy of a concept with regard to its intended function. When applied to conceptual engineering, and especially given its broad-spectrum ambitions as a metaphilosophical movement (Sect. 2), the most important of the three parameters is fruitfulness. The fruitfulness of a concept describes its suitability for formulating generalizations. For our specific purposes, it is measured in terms of the resulting scope and impact each of the two competing concepts of concept will grant conceptual engineering on our cognitive life (Sect. 3). As we will see, in both scope and impact, the psychological concept of concept outclasses the philosophical one.

In terms of scope, the issue at stake is whether the framework under consideration allows for an exhaustive or restrictive application of conceptual engineering to the variety of our conceptual devices. In this regard, the philosophical approach fares worse than the psychological one. With this approach, concepts are taken as semantic entities. They are, as such, primarily driven by truth requirements and further constrained by epistemic standards. In other words, when they enter, as unsaturated components, the formation of full thought contents, they are intended to provide us with true beliefs about their referent(s).^16^ Consequently, the philosophical concept of concept restricts the scope of items tractable by conceptual engineering to the most sophisticated ones only (that is, the theoretical concepts), to be re-engineered in accordance with standards proper to them (for instance, that of scientific rationality), so that they better serve their theoretical purposes (such as formulating true generalizations of explanatory value). Otherwise, were the scope of conceptual engineering broadened so as to include more mundane concepts, one would end up applying theoretical standards to them, building on the unwarranted assumption that there is a correspondence between the two (Carnap 1950: 8–11). And this would amount to nothing less than *metábasis eis állo génos* (cf. Strawson 1963). In such cases, indeed, mundane, non-theoretical concepts are usually treated as ‘prescientific’ (Carnap 1950: 1) glitches to fix so that they align better with the scientific image of the world (cf. Sawyer 2018, 2020a, b).^17^

By contrast, the psychological approach treats concepts as cognitive entities. As such, they are constrained mostly by pragmatic standards and ultimately driven by efficacy requirements. That is, when concepts are activated to play a role in the cognitive processes that underwrite the exercise of our higher cognitive competences, they are intended to enable the execution of the cognitive tasks we use them for, independently of any alethic or epistemic concerns.^18^ Consequently, the psychological concept of concept broadens the purview of conceptual engineering so that it ranges from the more mundane to the most sophisticated concepts, while at the same time respecting their diversity through the valuation of their functional efficacy according to multiple standards and purposes, ranging from the exposure and prevention of fallacies to the promotion of group agency or changes in group identities, via the alteration of people’s daily behaviors by nudging certain cues in their choice architecture, etc.^19^ Therefore, in terms of scope, the different outcomes for the philosophical and psychological options boil down to their relative, that is, more or less appropriate, calibration to conceptual diversity.

In terms of impact, the issue in question is whether the philosophical or psychological framework allows for a more direct connection with people’s minds and cognitive lives.^20^ The philosophical option also fares worse here because, in its view of concepts as semantic entities, concepts act as mere regulatory principles for the epistemic (viz., knowledge-related) processes and competences of some ideal epistemic agent. This becomes obvious when one considers what likely count among the most important desiderata for any philosophical theories of concepts, along with their corresponding explanatory purposes: On the one hand, the stability of content for explaining the agent’s rational/logical inference-making, as well as the ‘publicity’ or ‘shareability’ of concepts between agents; and, on the other hand, the compositionality of content for explaining the systematicity and productivity of the agent’s thought. In this light, philosophical theories of concepts may indeed only serve to formulate normative possession and application conditions for concepts that are primarily met, if not only, by agents who are both idealized and epistemic.^21^ As a consequence, the philosophical concept of concept threatens to turn conceptual engineering into the rather frivolous activity of fiddling with representational contents whose bearing on semantic facts is neither necessary nor sufficient to effect the desired changes in people’s cognitive abilities and behaviors, since these contents need not be grasped, fully, correctly, or even possibly, by those who actually do possess and use them.^22^ Therefore, the philosophical approach seemingly allows conceptual engineering to have only a very limited impact on our actual cognitive life.

By contrast, in the psychological view of concepts as cognitive entities, concepts operate as constitutive principles for the cognitive processes and competences of real cognitive agents. They are bodies of information about some category of referents that are activated, or constructed, to play a causal role in the processes that underlie the exercise of our higher cognitive competences. Characterized in this way, they serve psychological theories of concepts as *explanans* that contribute to producing empirical propositions about how our minds actually work. The psychological concept of concept thus turns the assessment and improvement phases of conceptual engineering into a twofold operation of first making explicit the typically opaque role that “concepts play in people’s cognitive life” (Machery 2017: 224), and second, of changing people’s minds and ways of thinking when they are found to be improvable. As a result, the psychological approach to conceptual engineering appears to be both necessary and sufficient to bring about the desired changes in people’s cognitive behaviors and abilities.^23^ Thereby, it arguably secures a strong impact for the method of conceptual engineering on the whole of our cognitive life. As Machery (2017: 231) expressed it: “Concepts determine the inferences we are prone to draw, and our thoughts follow their tracks. Understanding these inferences in order to assess them is one of the reasons why conceptual [engineering] matters.” In sum, the psychological concept of concept outclasses the philosophical one according to both criteria for measuring fruitfulness in our version of Carnapian explication repurposed for conceptual engineering—namely, in both scope and impact.

### Round Two: Unambiguity, Precision, and Consistency

Exactness is the second of our three parameters for assessing the efficacy of a concept with regard to its cognitive function. For our purposes, exactness has been characterized in terms of unambiguity, precision, and consistency (Sect. 3). Here as well, the psychological concept of concept outclasses the philosophical one. Let’s consider these three qualities in reverse order.

Consistency hinges on the absence of inconsistent constitutive principles that either contradict one another or conflict with otherwise uncontroversial facts and is detected by the presence of paradoxical issues. For instances of conceptual amelioration, change is indisputable, and the conundrum lies in having to guarantee some form of continuity through it. Otherwise, without any limits imposed on ameliorative projects in terms of continuity, they become trivial, as this would allow for arbitrary replacements/revisions (such as of WORLD WAR II with H_2_O). In brief, as Cappelen (2018: 97) notes: “We need to know: how much engineering is too much?”^24^

In this regard, the philosophical concept of concept fares worse than the psychological one because, as per its constitutive rationalist standards (Sect. 5.1), the former has to comply with a diachronic stability requirement for conceptual content. This requirement, which furthermore is meant to hold true ‘between-individual’ (Machery 2009: 14), conflicts *de facto* with the very possibility of conceptual change. Consequently, those who endorse a semantic approach to concepts for conceptual engineering end up resorting to some cumbersome notions and/or entities when they set out to address the ‘discontinuity objection’ and ensure the possibility that a concept changes while remaining the same, for instance, by talking of the ‘central function’ of concepts (Haslanger 2000), their ‘proper function’ (Thomasson 2020), and ‘essential features’ (Prinzing 2018), or by introducing ‘topics’ on top of concepts as their stable target (Sawyer 2018, 2020a, b). Since they are merely conceived to account for the effect they are meant to cause, all these strategies can quite rightly be discarded as ad hoc stipulations bereft of proper explanatory power.^25^

There is nothing comparable for those who endorse a cognitive take on concepts for conceptual engineering. Psychologically construed concepts also need to be stable, but “in a much more relaxed way” (Löhr 2018: 12)—that is, particularly, in a way that fits how our cognitive behaviors and abilities adapt to the complexities of the real world. For instance, in both their invariantist and contextualist versions, the stability requirement for psychological concepts of concept allows for the (overall) information that is activated in our concept-involving cognition to vary across context in accordance with the cognitive task to be executed (e.g., Machery 2015). Thus, what needs to be preserved in the psychological picture is only the causal/explanatory role that the concept plays in the processes that underlie the exercise of our higher cognitive competences. And such functional continuity of concepts, at work ‘within-individual’ only (Machery 2009: 14), can quite easily accommodate radical changes, because the (re-)engineered concept ultimately only needs to enable us to execute the cognitive task we used it for, regardless of diachronic or inter-agent discontinuities. Furthermore, contrary to its philosophical counterparts, the cognitive function of psychologically construed concepts has its own independent rationale—namely, to contribute to explaining how our minds work (Sect. 4).

The result is the same for precision, understood in terms of descriptive accuracy, with theoretical convergence as a possible marker. Indeed, the philosophical approach is infamous for having failed to identify the basic constitutive features of concepts. As Machery (2017: 234) notes, “most philosophers analyzing concepts by means of the method of cases have missed the fact that typically people do not form bliefs [*sic*, belief-like states] about necessary and sufficient membership conditions and the fact that typicality gradients are an important manifestation of concepts.” And “fail[ing] to hone in on such basic features” (ibid.) prevents the emergence of theoretical consensus around a well-supported model of concepts, as witnessed by the “smorgasbord of options for how to think about concepts” (Cappelen 2018: 141) semantically. By contrast, the psychology of concepts has witnessed many empirical advances over the years concerning the constitution of conceptual structures as being made up of specific bodies of information about some categories of referents (viz., individuals, classes, substances, event-types), along with the growing theoretical consensus on the compatibility of their different basic types (e.g., exemplars, prototypes, theories, etc.) at work in the processes driving the exercise of our higher cognitive competences (e.g., categorization, deduction, induction, analogy-making, action-planning, linguistic understanding, etc.). And these many empirical advances provide substantial ground for choosing the psychological approach over the philosophical one when it comes to theorizing concepts as the subject matter of an actionable method.

Lastly, in regards to unambiguity, which is understood in terms of descriptive adequacy with positive theoretical results as possible markers, the semantic approach is also ill-reputed for having failed to deliver any successful (positive) analysis of concepts *qua* definition-like structured entities—otherwise known in the literature as ‘Plato’s Problem’ (Margolis and Laurence 1999: 14). In contrast, the many empirical explanatory results about the mind’s higher cognitive competences produced and replicated in the psychology of concepts over the last 50 years (see footnote 15) provide additional reasons for choosing the psychological concept of concept over the philosophical one when it comes to theorizing about concepts as the subject matter of an actionable method. To sum up, given the bad track record of the semantic approach to concepts, along with the discontinuity conundrum, the philosophical concept of concept is again outclassed: Its psychological counterpart is superior when it comes to assessing its own efficacy with regard to exactness, which is characterized, in our rebooted Carnapian explication, by unambiguity, precision, and consistency.

### Round Three: Definition and Rules of Use

Simplicity is the third and last of our parameters for assessing the efficacy of a concept with regard to its cognitive function. Simplicity has been analyzed above into two components—namely, definition and rules of use. The definition component critically bear on the possibility of obtaining an operationalizable concept of concept, while the rules of use component depends on the usability of that definition to detect the need for conceptual amelioration and then to implement the prescribed changes (Sect. 3). In both respects, the psychological concept of concept outclasses the philosophical one.

Beginning with the issue of definition, and as a corollary of its failure to identify the basic constitutive features of concepts (Sect. 5.2), the semantic approach has failed to provide any operational characterization of concepts—that is, an articulated statement of procedures specifying how to detect the theoretical entities ‘concept’ in an experimental setting (including thought experiments). In contrast, the cognitive approach simply provides such an operational characterization of concepts, as a corollary of the theoretical convergence about its descriptive accuracy (ibid.). For instance, according to invariantist models, the bodies of information that constitute concepts are seen as being retrieved by default from our long-term memory to play a causal/explanatory role in the cognitive processes that underwrite our higher cognitive competences (cf. Sect. 4); and this defaultness condition can in turn be operationalized by a homeostatic cluster of three properties (namely, speed, automaticity, and context-independence), which itself enables the detection of concepts in various experimental settings (Machery 2015). As we turn to the issue of rules of use, the availability of such operational characterization, or lack thereof, thus directly fosters, or hinders instead, the usability of the definition under consideration for devising an actionable method for detecting the need for conceptual amelioration and then implementing prescribed changes.

In effect, as a corollary of its failure to deliver any successful conceptual analysis (Sect. 5.2), the semantic approach offers dim prospects for devising an actionable method to detect the need for conceptual amelioration. This methodological shortcoming has been clearly established on the basis of the substantial body of empirical evidence gathered over the last twenty years by the negative program in experimental philosophy against the reliance on intuitions, usually via the method of cases, for testing conceptual analyses and thereby ascertaining the actual content of concepts, which is crucial for detecting the need for conceptual amelioration (Sect. 2). According to Machery (2017: 234), in terms of ‘the poverty of the method of cases for conceptual analysis’: “The method of cases 1.0 is not suited for examining conceptual diversity; it is poorly suited to identify the weights of the properties represented by concepts and the interactions between these properties; its capacity to identify the properties represented by concepts is limited; and it often relies on disturbing cases.” Notwithstanding a few possible improvements, such methodological insufficiency disqualifies the semantic approach to concepts as a way to devise an actionable method for detecting the need for conceptual amelioration.

Furthermore, when it comes to then implementing prescribed conceptual changes, the semantic approach faces the following dilemma (Deutsch 2020a, b): Either the stipulative revisions/replacements prescribed are meant to operate at the individual level of speaker-meaning and speaker-reference, in which case conceptual engineering is reduced to triviality, for we do this all the time without even being aware of it; or these revisions/replacements target the communal level of semantic-meaning and semantic-reference, in which case conceptual engineers owe us an account of how to scale up from the individual level to the collective level and actually secure uptake for the prescribed changes—besides the inscrutability and uncontrollability of the many various factors that presumably affect the determination of semantic-meaning and semantic-reference (Cappelen 2018).^26^ Therefore, with respect to both the evaluative and prescriptive components of conceptual engineering (Sect. 3), the semantic approach to concepts fares poorly.

By contrast, the cognitive approach once again proves much better on the two aspects of the rules of use specified above. First, in terms of the prospects it offers for devising an actionable method to detect the need for conceptual amelioration, the cognitive approach can already rely on a ready-to-use experimental method of cases which both overcomes the limitations of its armchair predecessor and has already proved its suitability for assessing the content and deployment of psychologically construed concepts. For instance, for understanding people’s ways of thinking about things and squaring them with alternative images of the world in order to see whether and where they match with one another or instead are at odds (Machery 2017: 232 ff.); for assessing the control competent thinkers have when it comes to reasoning with new concepts (Fischer 2020); or for testing the prospects of proposed ameliorations to be successfully implemented for a certain class of concepts (Machery forthcoming).^27^ More generally, the cognitive approach will easily avail itself of a wide range of proven procedures, models, and methods from the social and cognitive sciences, from psycholinguistic experiments to framing via cognitive semantics, because the relevant ones build on psychological models of concepts (e.g., as exemplars, prototypes, or theories) as their target objects.

When it comes to implementing the prescribed conceptual changes, the psychological concept of concept is once again promising. Typically, in an invariantist model (see above), conceptual changes can be implemented by targeting either the content or the use of our conceptual structures. In the former, we would aim to re-delineate the default bodies of information that underwrite the exercise of people’s higher cognitive competences about some category of referents by, for instance, changing relevant parameters in the physical environment they are frequently exposed to.^28^ In the latter, we would endeavor to mitigate the default deployment of people’s concept-underwritten inferences by, for instance, changing the usage pattern of a word.^29^ Neither of these options, to be sure, is trivial or impossible ( cf. Deutsch 2020a, 2021), as when the government’s health campaigns directed us, with mixed success, to enact the concept of social distancing by socializing distantly with others (by using Zoom, keeping two meters apart, wearing masks), instead of distancing ourselves socially from one another (that is, by not seeing and talking to anyone anymore), as would have naturally been expected from what is written on the tin (namely, ‘social distancing’). In sum, when it comes to simplicity analyzed in terms of definition and rules of use in our rebooted version of Carnapian explication, the psychological concept of concept surpasses the philosophical one in both regards.

### Assessment Summary

In every respect, the psychological concept of concept, rather than the philosophical one, proves to be the most conducive to the purposes of conceptual engineering. Moreover, it also spares conceptual engineering from being turned into something akin to Mr. Jourdain’s prose: A mere fancy label for something we have been doing all along—namely, conceptual analysis, modulo a normative bent.^30^ Consequently, it is the one that should be preferred in order to conceive of concepts as the subject matter of conceptual engineering and make it an actionable method, that is, a method capable of being effectively applied to specific case studies.

## Objections and Replies

In this final section, I shall consider and rebut three critical objections to my case for a psychological theorization of concepts as cognitive entities for use in conceptual engineering, all of which concern Sect. 4. They are addressed below in order of decreasing importance.

### The Strawman Objection

To begin with, with regard to my framing of the philosophical approach, one may object that the classical theory of concepts is now a strawman. To this, I would simply reply that, from Burge (1993) to Margolis and Laurence (2019), it is still part of the traditional view to construe concepts as being endowed with some kind of definitional structure. Moreover, I would add that, whatever the case may be, it remains that the definitionist model of concepts still plays a normative role in that it represents a kind of regulatory, non-constitutive ideal which “the ‘Standard Model’ of philosophical inquiry” (Nado 2020a: 2) must comply with.^31^ In this respect, I would assert that the classical theory of concepts remains instrumental, albeit covertly, in current mainstream philosophical methodology—some have even recently defended the suitability of the classical definitionism for conceptual engineering.^32^

Yet, one may then argue more subtly that, on account of its reliance on the classical model, my ‘philosophical concept of concept’ only concerns a subset of philosophical theories of concepts, namely, the Containment Model, based on an internalist understanding (cf. Rey 2010), and one may thus question whether my critique rightfully extends to the Inferential Model, as well as to externalist accounts, in both the Containment and Inferential Models (see footnote 14). Here, my response would be that seemingly both the Containment and Inferential Models, in both their internalist and externalist forms, match my general description of the philosophical concept of concept, insofar as they all attempt to account for the conditions under which ideal epistemic agents can have propositional attitudes about the object of their attitudes, and thereby deliver “truths about the world” (Machery 2017: 209). And they may well do so, whether as applicability conditions, in the form of basic semantic features an object must satisfy in order to be included in a concept’s extension, as traditional internalist models would have it, or as reference-fixing conditions, in the form of “a higher-order test for determining what the correct applicability conditions are, without actually providing the applicability conditions themselves” (Schroeter 2004: 430), as in modern externalist-compatible models. But, in both cases, they radically differ from the project of explaining the processes that underlie our higher cognitive competences and thereby delivering “empirical propositions about the mind” (Machery 2017: 209). And I would point out that such epistemically responsive, truth-driven aims, which characterize the semantic approach in opposition to its cognitive counterpart, have been shown to work poorly for the purposes of conceptual engineering, especially when it comes to the scope and impact this approach grants conceptual engineering with regard to our conceptual apparatuses (Sect. 5.1). As a matter of fact, very few conceptual engineers are likely to be found aiming to make semantic changes an end in themselves, without care for “any non-semantic state of affairs obtaining” (Riggs 2019: 6).

I am willing to grant, however, that the Inferential Model can be seen as modeling the inferential patterns that concepts underwrite. But, in this case, I would first call for distinguishing between two versions of inferentialism: On the one hand, a semantic-epistemic version that sees concepts as underwriting inferential transitions that hold between the propositions they help forming and to which we are entitled; and, on the other hand, a psychological-cognitive variant that sees concepts as underwriting the exercise of our higher cognitive competences and to which we are reactive. I would then reject the possibility of understanding the inferential modeling either semantically or epistemically—that is, as articulating the concept’s meaning, or as tracking a certain type of information that has some kind of “distinctive justificatory status” (Machery 2017: 222) (see footnote 18), respectively—in favor of its psychological-cognitive construal, which is meant to capture “[the] inferences the mind is disposed to draw, that, so to speak, spring to mind, that it only resists when attention is drawn to particular facts that defeat this disposition” (Machery 2017: 222).

### The Dual Content Objection

Following upon the previous objection, one may contend that philosophical and psychological theories of concepts are not mutually exclusive, but instead compatible and complementary frameworks, dealing with “different aspects of the same kind” (Löhr 2018: 2).^33^ Notwithstanding the burden of having to explain in what sense concepts can be said to be ‘of a same kind’ (cf. Machery 2005, 2006, 2009; Piccinini and Scott 2006; Weiskopf 2009), I am perfectly willing to acknowledge that concepts characterized psychologically and philosophically share some common ground, however minimal: Both are types of intentional content. Yet, in the case of psychological theories of concepts, the bodies of information that constitute concepts are functionally characterized as cognitive entities, and such characterization is neither semantically nor epistemically based—for instance, distinctively, it does not build on the analytic/synthetic or on the a priori/a posteriori distinction. Therefore, the psychological take on concepts has no truck with attempts to determine the conditions under which ideal epistemic agents may have propositional attitudes about the objects of their attitudes, perhaps in the hopes of spelling out some “truths about the world” (Machery 2017: 209). It only cares about real people’s mind; and this, I assert, is what conceptual engineering should be all about: Changing people’s cognitive lives, abilities, and behaviors. As Nado (2020a: 4) states, cognitive efficacy is the chief aim of conceptual engineering; “truth and knowledge, by contrast, are secondary goals at best.”^34^ Thus, even if concepts were to be of a unique kind whose different aspects were theorized about by different, yet compatible and complementary, frameworks, the non-psychological one would not serve the purposes of conceptual engineering.

### The Concept-Conception Objection

In the same vein as the previous objection, one may eventually consider reframing the dichotomy between philosophical and psychological concepts of concept in terms of the distinction between concepts and conceptions, respectively (Rey 1983, 1985, 2010), and then assert, in accordance with Greenough (2020: <ms.>), that “<*Conception Engineering* is all we really need>” (see also Sawyer 2018, 2020c). However, this would be problematic. First, because ‘conception’ is a fuzzy philosophical notion, whose psychological counterpart can be, by contrast, clearly characterized and forms a well-established disciplinary field (namely, that of the psychology of concepts), with abundant, replicated, and widely accepted empirical results (see footnote 15); second, by the same token, because suggesting that the concept of concept should be restricted to its philosophical acceptation (with no further qualification) would give credence to the *cliché* of philosophy’s arrogant ignorance about empirical information (then relegated to concern conceptions only); and third, most importantly, because the concept-conception distinction builds on the assumption of the complementarity of both its components along with the ontological and epistemic prevalence of the former over the latter. Conceptions, indeed, typically correspond to the “inessential conditions associated with a concept” (Rey 1985: 302), which merely intimate its ascribability to a real cognitive agent, whereas concepts supposedly comprise the essential conditions that determine their individuation and possession by what looks like some kind of ideal epistemic agent (cf. Sect. 5). By contrast, when it comes to the psychological model advocated here, there are no other entities that concepts are associated with or dependent on. Therefore, recourse to the concept-conception distinction would be misguided.^35^

## Conclusion

This paper has focused on the theoretical foundations of conceptual engineering, which is characterized as the method used for assessing and improving our concepts. The main problem it has addressed is that of the subject matter of conceptual engineering, namely: How best to conceive concepts for the purposes of conceptual engineering. My strategy for tackling this problem has been to deploy an early version of conceptual engineering in order to target the concept of concept at work in ‘conceptual engineering.’ First, I established a provisional methodological framework against which to assess the conduciveness of a given concept of concept as subject matter for conceptual engineering (Sect. 3). Then, I distinguished two main competing concepts of concept that are available as subject matter for conceptual engineering—namely, the philosophical and psychological (Sect. 4). Finally, I assessed these two concepts of concept with the proposed methodological framework (Sect. 5). The main outcome of this assessment was that the psychological concept of concept beat its philosophical counterpart on all counts when it came to making conceptual engineering actionable, that is, a method that can effectively be applied to specific case studies. Therefore, for the purposes of conceptual engineering, concepts should be conceived psychologically as cognitive entities.

At this point, one final objection might arise that this paper itself acts as a counter-example to its assumption and thesis, namely, that we need an agreed-upon concept of concept for conceptual engineering, and, further, that this concept of concept should be construed psychologically. Fortunately, this objection is easy to rebut by simply denying its premise, namely, that, *strictly speaking*, the paper in itself constitutes a case study in conceptual engineering. And this is consistent with the prescriptive spirit of the paper, which aims to set the framework for future projects and research in conceptual engineering.

Now, once the philosophical framework and its semantic view of concepts has been dismissed in favor of a cognitive, naturalistic, and empirically informed one, the next step is to improve the psychological concept of concept further for the purposes of conceptual engineering. As noted in our provisional methodological framework (Sect. 3), the improvement phase of an engineering project must meet two requirements: First, the re-engineered concept has to maintain a certain degree of similarity with respect to the original target concept—in this case, by preserving the explanatory function of the concept of concept for the cognitive functionalism that characterizes conceptual engineering’s view of our concepts as cognitive devices (Sect. 2); and second, the re-engineered concept has to score better on each of the three assessment phase parameters. These two requirements are already satisfied by the psychological concept of concept by virtue of its selection via comparative assessment with its philosophical counterpart. Yet, this selection goes further: It also provides us with a baseline from which the psychological concept of concept can be further improved for the purposes of conceptual engineering. For instance, in order to increase the scope and impact conceptual engineering may have on our conceptual apparatuses, future research in this direction could appropriately draw upon a psychological, invariantist characterization of concepts as bodies of information that are retrieved from our long-term memory to play a role in the cognitive processes that underlie our higher cognitive competences, and then combine it with a pluralist take on the basic kinds of bodies of information (e.g., exemplar, prototypes, and theories) in the form of a model of concepts as “multiply realiz[ed] functional kinds” (Lalumera 2010: 218). However, this remains another story to be told.

## Footnotes

1 For some noteworthy exceptions, see Isaac (2020) , Koch (2020a), Machery (2017), Nefdt (2021), Scharp (2013).

2 See Isaac (2021b) on why conceptual engineering should be about concepts, instead of any other representational devices (linguistic meanings, intensions, speaker-meanings, etc.), on pain of pragmatic inconsistencies otherwise.

3 For example, see Brigandt and Rosario (2020), Brun (2016), Burgess and Plunkett (2013a), Burgess and Plunkett (2013b), Cappelen (2017), Cappelen (2018), Cappelen and Plunkett (2020), Chalmers (2020), Dutilh Novaes (2020b), Eklund (2014), Floridi (2011), Haslanger (2020), Machery (2017), Nado (2020a), Plunkett (2015), Plunkett (2016), Prinzing (2018), Scharp (2020), Simion (2018b), Thomasson (2020), among many others.

4 The notion of “function” is used here in its ordinary sense, without any substantial metaphysical component, and rather as that which simply qualifies a device *qua* device—that is, as something that has been made and/or is used to fulfill some purpose. Presumably, every conceptual engineer agrees about framing concepts in this way. Hence the widespread use of the word ‘device’ in the literature.

5 One might think that, in such case, there’s no need to start by assessing the actual or intended function of a given conceptual device. But, that would be a mistake, because diagnosing the need for revising or replacing a detrimental function of some concepts presupposes having identified and established beforehand the detrimental aspect(s) of that function (cf. Haslanger 2012: 223–224). Otherwise, one might end up worsening the functionality of something that was perfectly functional, resulting in some sort of *Verschlimmbesserung* (‘disimprovement’) then. As Thomasson (2020: 454) warns, “conceptual engineering, no less than civil, does not take place in a vacuum.” Thanks to an anonymous reviewer for pressing me on this point.

6 The following caveat seems appropriate here, formulated however in terms of analysis (instead of engineering): “[I]n practice conceptual analysis is often partly descriptive and partly prescriptive, and it is also often difficult to identify which component of an analysis is descriptive, and which is prescriptive: Analysis often describes the content of a concept and regiments it in one stroke” (Machery 2017: 217). Note that the importance of preliminary descriptive work is often overlooked in the literature that deals with conceptual engineering *qua* philosophical method. In addition to Machery (2017), other exceptions include Burgess and Plunkett (2013b), Dutilh Novaes (2016), Eklund (2021), Nado (2021), Plunkett (2016), Thomasson (2020), as well as some whose work reframes Carnapian explication as a precursory form of conceptual engineering (e.g., Brun 2016; Dutilh Novaes 2020a; Koch 2019; Schupbach 2017; Shepherd and Justus 2015).

7 As Nado (2020a: 17) puts it: “[A] concept is successful if it effectively fulfills its functions—in short, if it is *effective*. We could say, then, that the goal of conceptual engineering is to design effective concepts […]. Success is measured in terms of efficacy.”

8 See Brun (2016, 2020), Cordes (2020), Dutilh Novaes and Reck (2017), Dutilh Novaes (2020a), Pinder (2017, 2020), Schupbach (2017), Shepherd and Justus (2015) on how to overcome the limitations of Carnapian explication in the spirit of conceptual engineering.

9 This allows for divergences from the original framework. In particular, instead of primarily serving explanatory theoretical purposes, our rebooted version of Carnapian explication will aim to better fit the cognitive quality of the target concept to its intended function in terms of efficacy (Sect. 2)—namely, that of the concept of concept for the purposes of conceptual engineering. Thanks to an anonymous reviewer for pressing me on this matter.

10 The bootstrapping metaphor was originally coined for a series of self-sustaining processes to better oneself that proceeds without external help; here, it is meant to illustrate the recourse to a precursory variant of conceptual engineering (viz., Carnapian explication) in order to engineer the concept of concept in ‘conceptual engineering.’

11 Note that, given the importance of the so-called ‘implementation challenge’ and other ‘feasibility constraints’ for projects in conceptual engineering (Cappelen and Plunkett 2020; Deutsch 2020a, 2021; Fischer 2020; Jorem 2020; Koch 2020b; Machery forthcoming), and *pace* (Carnap 1950: 7), who takes it to be of ‘secondary importance,’ simplicity presumably overrules exactness, at least at times, when it comes to achieving conceptual changes. As already noted, our rebooted version of Carnapian explication might here radically diverge from the original framework (cf. footnote 9).

12 For a compendium of views on these controversies, see the commentaries in Machery (2010).

13 For example, see Brun (2016), Burgess and Plunkett (2013a), Chalmers (2011), Haslanger (2020), Plunkett (2015, 2016), Prinzing (2018), Richard (2020), Simion (2018a), Simion and Kelp (2020), Thomasson (2020), among others.

14 Two addenda to this basic characterization of the classical model (and its variants) are in order: First, “it’s natural to construe [the complex structure of concepts] in accordance with the Containment Model, where the components of a complex concept are among its proper parts. <It’s natural, but not mandatory. Alternatively, one could think of a classically structured concept as a node that stands in inferential relations to its defining features [viz., in accordance with the so-called ‘Inferential Model’]. The advantage of the Containment Model is that it makes especially clear which associated concepts are its defining features and which are incidental>” (Margolis and Laurence 1999: 9 <footnote 10>). Second, so-called ‘neo-classical’ views either drop or modulate the sufficiency requirement for the constitutive conceptual features (Margolis and Laurence 1999: 52 ff.).

15 See Hampton (1997), Komatsu (1992), Goldstone et al. (2018), Machery (2009), Medin (1989), Murphy (2004), Prinz (2002), Smith and Medin (1981), among others, for detailed reviews of the main theoretical paradigms in the literature on the psychology of concepts.

16 In the literature on conceptual engineering, Simion (2018a: 923) clearly articulates this view: “Concepts, just like beliefs, are representational devices, their function is an epistemic one: to represent the world. In virtue of this function, concepts will be properly functioning when responsive to epistemic reasons, and malfunctional when responsive to practical reasons. Concepts will be good concepts qua concepts when they are epistemically good.”

17 Think for instance of the zealous biologist who would want you to revise or replace your lay concept of berry, the one you use at the fruit stall, in accordance with the actual scientific taxonomy, which classifies bananas and eggplants in the berry category, but excludes strawberries, blackberries, etc. See also Isaac (2021a), McKenna (2018), Podosky (2018), Simion (2018a, 2018b), for why epistemic standards may stand in the way of ameliorative projects about social kind concepts, both in terms of constructionist and realist ontologies.

18 As Machery (2017: 222) states: “The class of concept-underwritten inferences is distinguished in psychological terms, exactly as is the class of concept-constitutive bliefs [*sic*, belief-like states]. It is neither semantic nor epistemological. The class of concept-underwritten inferences neither articulates the semantic content of the concept (its meaning), nor does it have any distinctive justificatory status.”

19 More prosaically, to follow up with a comparison to the previous example (footnote 17), the standards will vary depending on whether you are drawing up botanical taxonomies in biology class or searching for a tasty ingredient to top up your crumble recipe.

20 One might wonder whether characterizing impact in terms of the closeness of connection to people’s minds and cognitive life does not unduly privilege the psychological option. Given that impact so construed builds on the general characterization of conceptual engineering (Sect. 2), such privileging would instead confirm the greater intrinsic conduciveness of the psychological concept of concept for the purposes of conceptual engineering. Thanks to an anonymous reviewer for pressing me on this point.

21 Burge (1993: 314–315) articulates this “‘traditional view’ of concepts” in exemplary fashion: “A metaphysically correct definition—one that states actual necessary and sufficient conditions, indeed essential or fundamental individuating conditions for instantiating a kind—need not be known, or knowable on mere reflection, by someone who has the concept. […] The meaning of a term [viz., the concept] at a given time is fixed by what an ideally reflective speaker would articulate by reflecting on all his intuitions, beliefs, dispositions to apply a term, and so on, with no reliance on advances in non-linguistic knowledge.”

22 Think for instance of how the meaning of the term ‘marriage’ has been expanded to include same-sex unions: If no one grasps it, marriage equality activism will not have achieved its goal (non-sufficiency condition); on the other hand, if belief-like states about same-sex unions come to underlie people’s use of the term, changes in its meaning seem to be supererogatory (non-necessity condition).

23 To follow up on the ‘marriage’ example above (footnote 22): If belief-like states about same-sex unions come to underlie people’s use of the term, nothing else is seemingly required for it to be deemed a successful project in conceptual engineering (sufficiency condition); and, on the other hand, without such changes in people’s belief-like states, marriage equality activism will not have achieved its goal (necessity condition).

24 Note that what has come to be known in the literature as the ‘discontinuity objection’ (cf. Strawson 1963: 505–506) primarily affects ‘revisionary’ attitudes toward conceptual engineering, which aim to ameliorate some conceptual devices through their modification, while leaving rather untouched the ‘revolutionary’ camp, whose take on conceptual engineering amounts to replacing deficient concepts with better surrogate(s) *simpliciter*, regardless of continuity concerns, or at the least, with less concern for these (e.g., Burgess 2014; Scharp 2013, 2020).

25 Another option is to adopt a more permissive semantic approach that leans toward a cognitive take (e.g., in the vein of Brigandt’s [2010] ‘concept’s epistemic goal’) or to conceive of meanings as temporally extended, malleable, and plastic entities, at least when talking about socially constructed properties and kinds (Greenough ms.).

26 As Deutsch (2020a) convincingly argues, the implementation problem outstrips the so-called ‘externalist challenge’ to conceptual engineering (Koch 2018).

27 In the same spirit, see also Nicholas Shea and colleagues’ work on metacognition directed at concept dependability and its impact on various cognitive processes ( e.g., Shea 2019; Thorne 2021).

28 For example, achieving gender balance in the professional world could lead to modifying the content and structure of people’s profession concepts and thereby contribute to mitigating the stereotypical gendered biases in their categorization process and general behaviors about these categories of profession.

29 For example, psycholinguistic studies have shown that the automatic deployment of gender-related stereotypical inferences may be mitigated by the explicit marking of deviation from stereotypes (‘male secretary’) or salient uses via, e.g., contextual cues (‘scratches his beard’) (Fischer 2020; Fischer and Engelhardt 2017).

30 From *The Middle Class Gentleman* by Moliére (Act 2, Scene 4): “By my faith! For more than forty years I have been speaking prose without knowing anything about it, and I am much obliged to you for having taught me that.”

31 Nado (2020a: 2) aptly portrays the Standard Model: “Philosophers, so the story goes, are in the business of producing and testing conceptual analyses. And intuition, generally via the method of cases, is our primary source of evidence for the success (or more commonly, the failure) of an analysis.”

32 “Necessary and sufficient conditions fare poorly in capturing our actual concepts, but I see no reason to abandon them as the gold standard in conceptual engineering.” (Nado 2020b: 10, footnote 4)

33 *Mutatis mutandis*, this could be recast in terms of the ‘Dual content’ theories of concepts—hence the title of this subsection.

34 See Sterken’s (2020) ‘linguistic interventions’ as ‘transformative communicative disruptions’ and Cantalamessa’s (2019) ‘conceptual activism,’ and Isaac’s (2021a) ‘post-truth conceptual engineering’ for paradigmatic instances of non truth-driven conceptual engineering.

35 See Lalumera (2014) for a more positive appraisal of the value of the concept-conception distinction.
